# 芦可替尼联合维奈克拉及阿扎胞苷治疗伴JAK1、JAK3、STAT5B基因突变的难治性T-ALL患者1例报告并文献复习

**DOI:** 10.3760/cma.j.cn121090-20240412-00138

**Published:** 2024-09

**Authors:** 佩佩 许, 彤 周, 岳一 徐, 苗新 彭, 颖 杜, 婷 谢, 永公 杨, 建 欧阳, 兵 陈

**Affiliations:** 南京大学附属鼓楼医院血液内科，南京 210008 Department of Hematology, Nanjing Drum Tower Hospital, Affiliated Hospital of Medical School, Nanjing University, Nanjing 210008, China

## Abstract

难治性急性T淋巴细胞白血病（T-ALL）在常规诱导缓解过程中对药物敏感性低、整体预后差。南京大学附属鼓楼医院报道了1例伴JAK1、JAK3、STAT5B基因突变的难治性T-ALL患者。标准VDLP（长春地辛、柔红霉素、培门冬酶、地塞米松）方案诱导化疗1个疗程后患者疾病未缓解，调整治疗方案为芦可替尼联合维奈克拉及阿扎胞苷。经治疗后，患者骨髓微小残留病（MRD）转阴，同阶段予以地塞米松联合依托泊苷胸腔灌注治疗后，患者胸腔积液及纵隔肿块较前明显好转。该患者骨髓缓解后行异基因造血干细胞移植，随访至移植后17个月，病情持续缓解。芦可替尼联合维奈克拉及阿扎胞苷治疗伴有JAK1、JAK3、STAT5B突变的难治性T-ALL疗效及安全性良好，为此类患者的治疗提供了新思路。

## Abstract (en)

Refractory acute T-lymphoblastic leukemia（T-ALL）, which is characterized by a low sensitivity to conventional induction therapy and poor prognosis, poses significant challenges during treatment. This study reported a case of refractory T-ALL patient with mutations in the JAK1, JAK3, and STAT5B genes from Nanjing University's Gulou Hospital. Following an unsuccessful course of standard VDLP regimen chemotherapy, the treatment was modified to include ruxolitinib in combination with venetoclax and azacitidine. Subsequent to this therapy, the patient achieved bone marrow minimal residual disease（MRD）negativity. Notably, pleural effusion and mediastinal mass significantly improved the post-chest cavity infusion of dexamethasone combined with etoposide at the same stage. The patient also underwent allogeneic hematopoietic stem cell transplantation upon achieving bone marrow remission and was followed up until January 2024. Ruxolitinib combined with venetoclax and azacytidine has shown promising efficacy and safety in treating refractory T-ALL harboring the JAK1, JAK3, and STAT5B mutations, providing a novel therapeutic approach for such patients.

急性T淋巴细胞白血病（T-ALL）是起源于淋巴母细胞的高度侵袭性血液系统肿瘤[1]，临床常伴有纵隔、胸膜侵犯及肝脾淋巴结肿大。成人T-ALL由于原发耐药以及早期复发等原因常致预后不良，5年无事件生存（EFS）率仅为20％～50％[2]。针对复发难治T-ALL的治疗手段有限，有效的靶向药物和免疫治疗策略尚在探索之中，故目前仍是以高强度化疗和异基因造血干细胞移植为主。本文报道了一例伴JAK1、JAK3、STAT5B基因突变的难治性T-ALL患者的临床诊治过程，并对相关文献进行复习，以期为难治性T-ALL的治疗提供新策略。

## 病例资料

患者，男，25岁，因“咳嗽10余天”于2022年4月15日入住我院。入院查体：颈部、锁骨上、腋窝、腹股沟可触及多个肿大淋巴结，最大淋巴结约为4 cm×2 cm，质韧。右肺呼吸音减低，脾脏触诊在左肋缘下7 cm。血常规：WBC 10.5×10^9^/L，淋巴细胞占67.5％，HGB 149 g/L，PLT 99×10^9^/L。胸部CT：上纵隔见软组织影，右侧大量胸腔积液伴肺膨胀不全。颈部淋巴结穿刺病理：条索样淋巴结示形态较一致的小淋巴细胞弥漫增生，隐约可见呈结节样，免疫标记表达部分T细胞标志及原始造血干细胞标志，考虑T淋巴母细胞淋巴瘤/白血病；免疫组化：肿瘤细胞CD3（+++）、CD8（+）、CD7（+++）、Ki67（90％+）、TdT（+++）、CD99（+++）、CD34（+++）；原位杂交：EBER（−）。为进一步诊治收入我院。骨髓相关检查：细胞形态学：原始淋巴细胞82％。流式细胞术免疫表型分析：占有核细胞46％的异常幼稚细胞群体表达CD7^bri^、cCD3^bri^，部分表达CD5、CD2、CD19、CD13、CD8、cCD79a、cTDT、CD22。染色体核型：46，XY。FISH：未检测到t（9；22）形成的BCR::ABL融合基因。基因突变二代测序：JAK3、JAK1、STAT5B基因突变频率分别为39.8％、10.4％、44.0％。未检出Ph-like基因。胸水相关检查：胸水常规：有核细胞计数为19 334.0×10^6^/L，淋巴细胞计数为15 647.0×10^6^/L，蛋白阳性。流式细胞术分析：异常幼稚T淋巴细胞群体占白细胞的75.05％，表达cCD3、CD7^bri^，部分表达CD34。临床诊断为“T淋巴细胞白血病伴B系和髓系抗原表达（WHO2016标准[3]）合并纵隔及胸膜侵犯”。

入院后行VDLP（长春地辛、柔红霉素、培门冬酶、地塞米松）方案诱导化疗。第1个疗程后复查骨髓细胞形态学：原始淋巴细胞65.5％。考虑为ALL未缓解（NR）。遂于2022年5月起行芦可替尼（10 mg，每日2次）+维奈克拉（100 mg每日1次联合泊沙康唑预防真菌感染）+阿扎胞苷（75 mg·m^−2^·d^−1^×7 d）方案治疗。1个疗程后复查骨髓细胞形态学：ALL-完全缓解（CR）。骨髓流式细胞术分析：T-ALL微小残留病（MRD）阴性。骨髓细胞二代测序：JAK1、JAK3、STAT5B等突变基因均转阴。6月至8月行第2、第3个疗程芦可替尼联合维奈克拉及阿扎胞苷治疗。期间行地塞米松10 mg单药胸腔灌注4次、地塞米松10 mg联合依托泊苷0.2 g胸腔灌注2次。8月复查胸部CT：胸腔积液较前明显较少，纵隔肿块直径变小。同时胸水细胞学及流式细胞术均未检测出异常细胞。PET-CT：颈两侧、左锁骨下、纵隔、右心膈角、腹腔及腹膜后多发淋巴结部分肿大（最大淋巴结大小约2.2 cm×1.5 cm），代谢均升高，SUVmax＝11.7。患者于2022年8月予以环磷酰胺（CY）+全身照射（TBI）预处理后行单倍体异基因造血干细胞移植（母供子）。移植后1个月复查骨髓细胞形态学：ALL-CR。骨髓流式细胞术：T-ALL MRD阴性。骨髓细胞二代测序JAK1、JAK3、STAT5B等突变基因均阴性。STR：供者细胞99.13％。PET-CT：淋巴结较前缩小、代谢减低。2023年3月起（移植后半年余）每月继续予以阿扎胞苷100 mg×5 d维持治疗。移植后17个月复查骨髓及PET-CT均正常，骨髓呈持续CR状态。

## 讨论及文献复习

难治性T-ALL在常规诱导缓解过程中对药物敏感性低、整体预后差，且再次诱导缓解率低，3～5年总生存（OS）率小于10％，中位生存时间仅有2～6个月[4]。目前对于难治性T-ALL，尚未有公认有效的化疗方案，异基因造血干细胞移植仍是治疗难治性T-ALL的最有效的手段。

本例患者经标准VDLP方案诱导治疗1个疗程后未缓解，通过应用芦可替尼联合维奈克拉及阿扎胞苷治疗后，成功使骨髓MRD转阴。芦可替尼作为JAK1/2抑制剂[5]，通过调控靶基因的表达，参与细胞增殖分化免疫调控过程从而抑制细胞的增殖[6]。目前临床上JAK激酶抑制剂，例如托伐替尼、巴瑞替尼等多用于免疫系统疾病[7]。在血液系统疾病中，芦可替尼多应用于骨髓纤维化、真性红细胞增多症、移植物抗宿主病中[8]–[9]。2008年Flex团队发现JAK1基因在淋巴样细胞前体增殖、存活和分化中发挥促进作用，该基因突变的ALL患者预后较差，对常规的ALL治疗方案具有耐药性，因此认为JAK抑制剂很可能成为新型靶向抗白血病药物[10]。Candan等[11]在2023年报道了1例JAK基因突变的Ph样ALL患者，经R-Hyper-CVAD及FLAG方案化疗后未缓解。随后联合芦可替尼治疗1个疗程后，患者获得CR。2014年Degryse等[12]证实JAK1的表达对于JAK3突变通过STAT5B信号转导至关重要（STAT5B是JAK2、JAK3的下游效应因子）。芦可替尼为JAK1、JAK2抑制剂，对JAK3作用微弱，但芦可替尼可以通过抑制JAK1，从而抑制JAK3及STAT5B信号转导抑制下游通路达到抗肿瘤作用。T-ALL患者因T淋巴细胞的异常表达，炎症因子水平常高于其他血液系统疾病的患者，多种炎症细胞因子可通过JAK-STAT通路进行信号转导。芦可替尼作为广谱的细胞因子抑制剂，可通过抑制免疫细胞产生的多种细胞因子，从而影响细胞凋亡、分化、细胞周期等多种生理和病理过程，使JAK1、JAK2介导的炎症细胞因子水平下调，起到降低细胞因子的作用[8]，[13]–[14]。芦可替尼还可以通过降低中性粒细胞迁移并抑制其刺激T细胞的能力，减少T细胞及中性粒细胞数量，抑制活化状态及组织浸润[15]，从而降低T-ALL患者的炎症指标，缓解病情。

BCL-2抑制剂维奈克拉通过结合BCL-2蛋白的BH3结构域，从而释放促凋亡蛋白，诱导细胞凋亡[16]，临床上常用于慢性淋巴细胞白血病、急性髓系白血病（AML）等。一些临床前研究结果提示，维奈克拉可能对早期前体T-ALL、Ph阳性及Ph样ALL有效[17]–[18]。2013年Kontro等[19]曾提出STAT家族中STAT3和STAT5基因的异常激活与血液系统恶性肿瘤的发生存在密切关系的观点，该研究表明，STAT5B对BCL-2抑制剂敏感。同年Vainchenker等[20]也提出STAT5参与造血分化，同时有加强干细胞的自我更新的能力。STAT5通过tad依赖机制诱导生长相关基因c-Myc、BCL-2和BCL-X的表达[21]–[22]。STAT5B突变在T-ALL中并不常见，且对靶向JAK激酶抑制剂不敏感。2021年Jabbour团队将维奈克拉应用于47例复发难治的ALL患者，CR率达到60％[23]。因此BCL-2抑制剂可能为T-ALL患者的新型候选疗法，为难治性T-ALL的治疗中提供了新的治疗思路。

2018年Karjalainen等[24]经实验证明在AML模型中芦可替尼和维奈克拉之间具有协同作用。Brothers等[25]在2023年报道了一例JAK1/3突变的复发T-PLL患者，在维奈克拉单药治疗无效后加用芦可替尼获得部分缓解。维奈克拉联合阿扎胞苷用于血液系统肿瘤方面的研究常见于AML及骨髓增生异常综合征[26]。2021年Wan等[27]纳入了5例难治复发高危T-ALL患者（3例T-ALL，2例ETP），均在维奈克拉联合阿扎胞苷治疗1个疗程后达到CR，联合用药耐受性良好。2022年高升等[28]报道了1例应用维奈克拉联合阿扎胞苷治疗复发的弥漫大B细胞淋巴瘤继发AML患者，经4个周期治疗后，患者复查CT未见增大淋巴结，淋巴瘤得到控制。由此可见维奈克拉联合阿扎胞苷对髓外肿瘤累及具有治疗作用。目前维奈克拉联合阿扎胞苷对ALL髓外受累患者的治疗效果，暂无报道。本例患者在予以芦可替尼联合阿扎胞苷及维奈克拉治疗后，骨髓达到CR，但髓外病灶未完全消除，一方面不排除药物使用剂量未达到髓外缓解的浓度的可能，另一方面考虑该患者疾病复发难治的特性。因此我们在预处理方案中予以地塞米松联合依托泊苷胸腔灌注，行局部化疗。患者在异基因造血干细胞移植后，多次复查PET-CT提示代谢转阴。目前临床上尚未有芦可替尼联合维奈克拉及阿扎胞苷治疗难治性T-ALL的相关报道。该方案对髓外肿瘤浸润的作用强度暂不明确，因此方案为JAK1、JAK3、STAT5B突变的患者提供了新的诊疗思路，同样也需要更多的临床研究对其进行进一步改进。

T-ALL的患者常伴有纵隔肿块及大量胸腔积液，行胸腔闭式引流缓解症状的同时予以胸腔灌注化疗药物，可以提高胸膜内药物的浓度，增强抗癌药物对肿瘤细胞的杀伤毒性，减少全身毒性。分析既往应用依托泊苷胸腔注射治疗癌性胸水的患者[29]–[30]，此项治疗多用于肺癌、乳腺癌、食管癌等癌肿侵犯胸膜导致的胸腔积液，其中血液系统肿瘤目前仅有两例淋巴瘤的报道。但应用于治疗ALL所致的纵隔大肿块及大量胸腔积液，为国内首例。依托泊苷作用于DNA拓扑异构酶Ⅱ活性，阻碍DNA的修复从而实现抗肿瘤的效果，还通过处理体内树突状细胞介导的T淋巴细胞IFN-γ反应的能力从而达到抗炎性风暴的作用[31]。而地塞米松磷酸钠与化疗药物联用时可提高肿瘤治疗效果，降低化疗药物的不良反应，显著抑制肿瘤生长和转移[32]，同时可发挥抗炎、抗血管生成作用。经治疗后患者胸腔积液得以控制，纵隔肿块明显缩小，代谢降至正常。

综上所述，在标准VDLP方案对难治性T-ALL疗效不佳的情况下，本例患者使用芦可替尼联合维奈克拉及阿扎胞苷治疗取得显著疗效，这为难治性T-ALL的治疗提供了新思路。随着免疫学、遗传学及分子生物学技术的进步，对T-ALL的发病机制的研究更为深入，靶向治疗的使用有望提高难治性T-ALL患者的疗效。
